# Expression of vascular cell adhesion molecule-1 in the aortic tissues of atherosclerotic patients and the associated clinical implications

**DOI:** 10.3892/etm.2015.2540

**Published:** 2015-06-03

**Authors:** WEI MU, MINGYOU CHEN, ZUSHUN GONG, FEI ZHENG, QICHONG XING

**Affiliations:** Department of Cardiology, Shandong Provincial Qianfoshan Hospital, Shandong University, Ji'nan, Shandong 250014, P.R. China

**Keywords:** atherosclerosis, coronary heart disease, vascular cell adhesion molecule-1, human aorta, coronary artery bypass graft

## Abstract

The aim of this study was to investigate the expression level of vascular cell adhesion molecule-1 (VCAM-1) in the aortic tissues of atherosclerotic patients and to explore the associated clinical implications. Full-thickness aortic wall tissue samples were collected from atherosclerotic patients. Biochemical analysis was used for the detection of the serum levels of triglycerides (TG), total cholesterol (TC), low-density lipoprotein cholesterol (LDL-C), high-density lipoprotein cholesterol (HDL-C), lipoprotein (a) [Lp (a)], apolipoprotein (Apo) AI and Apo-B. Coronary angiography and SYNTAX scoring were used to determine the extent and severity of the disease. Immunohistochemistry was employed for the detection of the VCAM-1 protein expression levels in the arterial tissues. Significant differences were observed in the blood lipid levels between atherosclerotic patients and control subjects. Immunohistochemistry indicated that the aortic VCAM-1 expression level in atherosclerotic patients was 0.23±0.06 optical density (OD) units, which was significantly higher than that in the control subjects (0.08±0.03 OD units). In the atherosclerotic patients, the aortic VCAM-1 expression was positively correlated with the serum levels of TG (r=0.347), TC (r=0.469), LDL-C (r=0.463), Lp (a) (r=0.507) and Apo-B (r=0.384), while VCAM-1 and HDL-C were negatively correlated (r=-0.319). Furthermore, a higher SYNTAX score was accompanied by a higher VCAM-1 expression level (r=0.532), and an elevated aortic VCAM-1 expression was associated with certain cardiovascular risk factors. In conclusion, aortic VCAM-1 expression is associated with the severity of atherosclerosis and cardiovascular risk factors, indicating that VCAM-1 plays a role in the pathogenesis of atherosclerosis.

## Introduction

Atherosclerosis, a systemic disease that usually affects large- and medium-sized elastic and muscular arteries, is the underlying pathology of most cardiovascular diseases. The onset and pathogenesis of atherosclerosis have not yet been fully established. Studies have concluded that the activation of the inflammatory pathways may be important in the pathogenesis of the disease (1,2). It has been shown that atherosclerotic risk factors can cause vascular endothelial injuries, inducing the endothelial expression of adhesion molecules, such as vascular cell adhesion molecule-1 (VCAM-1), and subsequently initiate inflammatory responses (3,4).

VCAM-1 belongs to the immunoglobulin superfamily, which is expressed in vascular endothelial cells. VCAM-1 promotes the adhesion of leukocytes to endothelial cells (5), and accelerates the migration of the leukocytes along the endothelial surface. In addition, VCAM-1 has been linked with the pathogenesis of atherosclerosis. De Caterina *et al* (6) showed that soluble VCAM-1 (sVCAM-1) levels were directly associated with carotid intima-media thickness and could be used to evaluate prognosis. Zeitler *et al* (7) also found that sVCAM-1 levels were significantly elevated in patients suffering from coronary heart disease and acute myocardial infarction. Although these findings concerning the role of sVCAM-1 in coronary heart disease are encouraging, the sVCAM-1 level only represents the proteins expressed on cell surfaces that are shed into the blood. It is therefore of great importance to investigate the expression levels of VCAM-1 in arterial tissues, and to elucidate the association between arterial VCAM-1 expression and the disease pathogenesis. Based on this, the aim of the present study was to investigate the expression levels of VCAM-1 in the aortic tissues from patients undergoing coronary artery bypass graft (CABG) surgery for coronary heart disease, and to explore the association between VCAM-1 expression and the pathogenesis of atherosclerosis.

## Materials and methods

### 

#### Patients

Thirty-four patients undergoing CABG [26 males and 8 females, aged 48–76 years (mean, 62±7 years)] were included in the study; all patients had been admitted to the Shandong Provincial Qianfoshan Hospital (Ji'nan, China) between December 2008 and February 2012. In the present study, indications for CABG surgery included left main lesions or bifurcation lesions insensitive to medical treatment, severe proximal left anterior descending artery stenosis, three-vessel disease, particularly when accompanied by cardiac dysfunction or diabetes mellitus, and intervention failure. The exclusion criteria were as follows: Any type of cancer, liver and/or kidney dysfunction, and chronic infectious, autoimmune, acute cerebrovascular and peripheral vascular diseases. Following admission, the patients received conventional anti-atherosclerotic treatment. A detailed medical history, including details of the present illness, past illnesses and family history, was completed, and physical examination and routine laboratory tests were carried out in order to establish a clinical diagnosis. Special attention was paid to cardiovascular risk factors, including smoking status, hypertension and diabetes mellitus. Out of the 34 patients, 18 patients were smokers, 20 had hypertension and 11 had diabetes mellitus.

The control group consisted of renal artery specimens, which were collected from 12 kidney transplant donors. As indicated by comprehensive physical examinations, these kidney transplant donors were free from organic diseases and did not have a history of coronary heart disease, hypertension or diabetes mellitus. Furthermore, all control subjects were non-smokers and without long-term medication. Prior written and informed consent was obtained from every participant and the study was approved by the Ethics Review Board of the Shandong Provincial Qianfoshan Hospital.

#### Biochemical determination

The morning after admission, 6 ml venous blood was collected from each subject in a fasting state. The colorimetric endpoint method was used to determine the levels of serum triglycerides (TG) and total cholesterol (TC), and the chemical modification-enzymatic method was used to detect the serum levels of low-density lipoprotein cholesterol (LDL-C) and high-density lipoprotein cholesterol (HDL-C). The levels of lipoprotein (a) [Lp (a)], apolipoprotein (Apo) AI and Apo-B were measured by immunoturbidimetry. A MODULAR biochemical analysis system (Roche Diagnostics AG, Basel, Switzerland) was used for the aforementioned analyses.

#### Coronary angiography and SYNTAX scoring

Coronary angiography was performed via the right femoral artery using the Judkins technique (8). The lesions were directly exposed, usually in the 45° left anterior oblique and 30° right anterior oblique projections, in order to perform left and right coronary angiography. During the coronary angiography, the complexity of the coronary artery disease was determined by the synergy between percutaneous coronary intervention with Taxus and the cardiac surgery (SYNTAX) score. From the baseline diagnostic angiogram, each coronary lesion producing ≥50% diameter stenosis in vessels of ≥1.5 mm was scored separately and added together to provide the overall SYNTAX score. The SYNTAX score was calculated using dedicated software (version 2.11; www.syntaxscore.com) (9).

#### Sample preparation and immunohistochemistry

Full-thickness aortic wall tissue samples were collected from the patients during the CABG surgery, and control renal artery tissues were obtained from kidney transplantation cases. The sample preparation and immunohistochemistry protocols were in accordance with those described in a previous study (10). Briefly, the samples were fixed in 10% formalin. Following alcohol dehydration and xylene clearing, the samples were embedded in paraffin and cut into slices. The sections were subsequently dewaxed with xylene and treated with citrate antigen retrieval buffer and 3% hydrogen peroxide. Rabbit anti-human VCAM-1 polyclonal antibody (1:300, bs-0920R; Beijing Biosynthesis Biotechnology Co., Ltd., Beijing, China) was used to incubate the sections at 4°C overnight. Horseradish peroxidase-labeled goat anti-mouse/rabbit immunoglobulin G conjugates were used for incubation for 30 min at room temperature. The peroxidase-diaminobenzidine reaction and hematoxylin staining were then performed. The sections were sealed with neutral resin and visualized with a fluorescence microscope. Three high-power fields were randomly selected from each slice and the average optical density (OD) was measured in each field.

#### Statistical analysis

Data are presented as the mean ± standard deviation. SPSS 13.0 software (SPSS, Inc., Chicago, IL, USA) was used to perform the statistical analysis. Following testing for the normal distribution of variables, the normally distributed continuous variables were analyzed with the Student's t-test. The correlation between two variables was determined by a simple regression analysis. A two-tailed P<0.05 was considered to indicate a statistically significant difference.

## Results

### 

#### Measurement of blood lipid levels in atherosclerotic patients

The serum lipid levels of 34 atherosclerotic patients are presented in Table I. As expected, the preoperative serum TG, TC, LDL-C, Lp (a) and (Apo) B levels in the atherosclerotic patients were significantly elevated, whereas the serum HDL-C and Apo-AI levels were significantly decreased, compared with the normal reference values (P<0.05) (Table I).

#### Expression levels of VCAM-1 in arterial tissues in atherosclerotic patients

**Figure 1.**
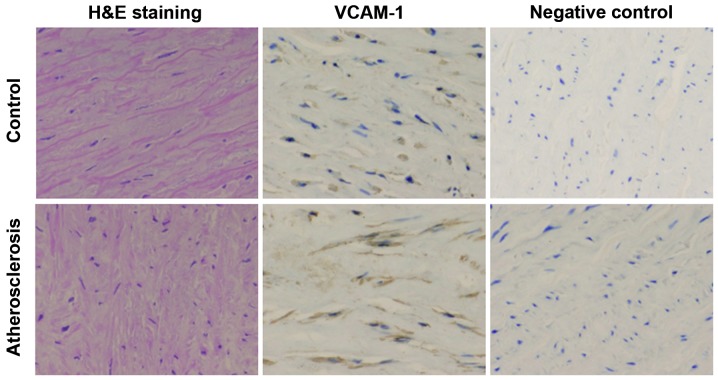
Immunohistochemical staining of VCAM-1 on arterial tissue sections. The expression level of VCAM-1 was increased in the arterial specimens from atherosclerotic patients (rabbit non-immune serum was used as a negative control) (original magnification, x400). VCAM, vascular cell adhesion molecule; H&E, hematoxylin and eosin.

To investigate the expression levels of VCAM-1 in the arterial tissues of atherosclerotic patients, immunohistochemistry was carried out. As shown in Fig. 1, brown staining of VCAM-1 was observed in the endothelial and smooth muscle cells. The expression level of VCAM-1 in the arterial tissues of the atherosclerotic patients was 0.23±0.06 OD units (range, 0.12–0.40 OD units), which was significantly higher than that in the control group tissues (0.08±0.03 OD units; range, 0.06–0.10 OD units). These results suggested that the expression levels of VCAM-1 in the arterial tissues were significantly elevated in atherosclerotic patients compared with those in the control subjects.

To further determine the association between the aortic VCAM-1 expression and the blood lipid indicators in atherosclerotic patients, correlation analysis was performed. The results showed that, in those patients, the expression levels of VCAM-1 in the aortic tissues were positively correlated with the serum levels of TG (r=0.347, P=0.046), TC (r=0.469, P=0.005), LDL-C (r=0.463, P=0.006), Lp (a) (r=0.507, P=0.002) and Apo-B (r=0.384, P=0.025), while a statistically insignificant negative correlation was observed between the aortic VCAM-1 expression and the serum HDL-C levels (r=-0.319, P=0.066). These results suggested that the elevated expression of VCAM-1 in the aortic tissues was associated with the pathophysiological changes in atherosclerosis.

#### Correlation between aortic VCAM-1 expression and coronary lesion severity in atherosclerotic patients

**Figure 2.**
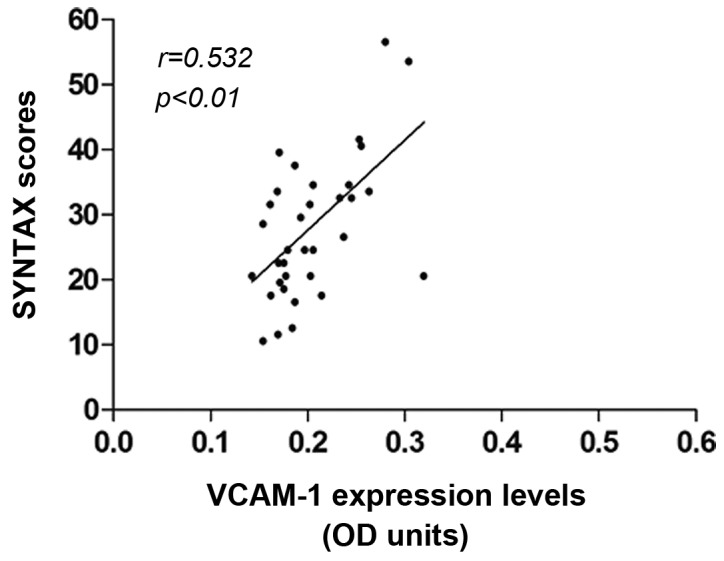
Association between aortic VCAM-1 expression and SYNTAX score in atherosclerotic patients. Correlation analysis showed that the SYNTAX score was positively correlated with the expression levels of VCAM-1 in the aortic tissues collected from the atherosclerotic patients. VCAM, vascular cell adhesion molecule; OD, optical density.

The correlation between the aortic VCAM-1 expression and the SYNTAX scores was investigated. The average SYNTAX score of the atherosclerotic patients was 29.35±13.26. The results indicated that higher SYNTAX scores were accompanied by higher VCAM-1 expression levels. Correlation analysis showed that the SYNTAX score (i.e., the lesion severity) was positively correlated with the VCAM-1 expression in atherosclerosis (r=0.532, P<0.01) (Fig. 2). These results suggested that the aortic VCAM-1 expression was linked with the severity of the coronary heart disease.

#### Association between aortic VCAM-1 expression and cardiovascular risk factors

**Figure 3.**
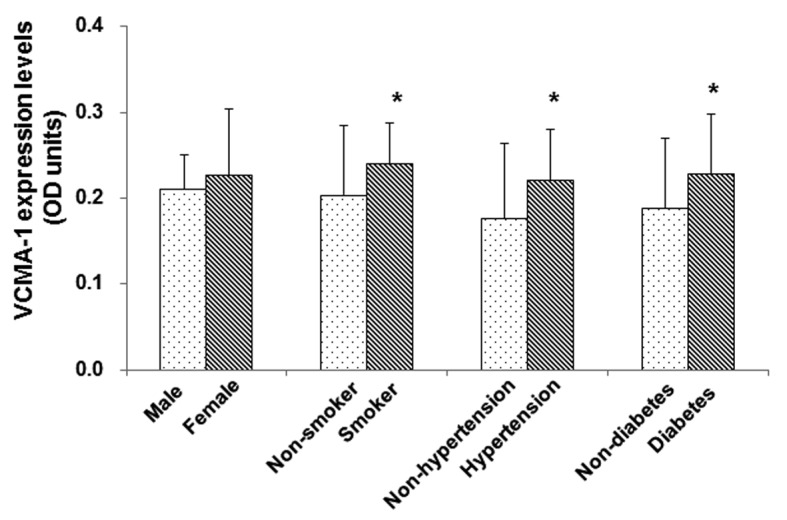
Association between aortic VCAM-1 expression and cardiovascular risk factors. Subgroup analyses were performed to investigate whether VCAM-1 expression would be affected by cardiovascular risk factors (gender, smoking, hypertension and diabetes) in atherosclerotic patients. Compared with the counterpart control, *P<0.05. VCAM, vascular cell adhesion molecule; OD, optical density.

The aim of the final investigation was to determine whether the expression of VCAM-1 in the atherosclerotic patients would be affected by certain cardiovascular risk factors. Subgroup analyses were performed based on gender, smoking, hypertension and diabetes mellitus, and the results showed that there were no significant differences in the aortic VCAM-1 expression levels between male (n=26) and female (n=8) atherosclerotic patients (0.21±0.04 vs. 0.22±0.07 OD units, P>0.05); however, the aortic VCAM-1 expression levels in smokers were significantly higher (n=18, 0.24±0.05 OD units) than those in non-smokers (n=16, 0.20±0.08 OD units) (P<0.05). Furthermore, the aortic VCAM-1 expression levels were significantly higher in atherosclerotic patients with either hypertension (n=20, 0.22±0.06 OD units) or diabetes mellitus (n=11, 0.23±0.07 OD units), compared with those in non-hypertensive (n=14, 0.18±0.09 OD units) and non-diabetic (n=23, 0.19±0.08 OD units) patients, respectively (P<0.05) (Fig. 3). These results indicated that major cardiovascular risk factors, such as smoking, hypertension and diabetes mellitus, are associated with the elevated aortic VCAM-1 expression levels in patients with atherosclerosis.

## Discussion

Cardiovascular and cerebrovascular diseases caused by atherosclerosis have become the leading cause of mortality in humans. Although the mechanism underlying the development of atherosclerosis is not yet fully understood, considerable evidence shows that inflammation is involved in the occurrence and pathogenesis of the disease, including inflammatory cell adhesion and migration and smooth muscle cell proliferation (4). Studies have shown that, with the stimulation of inflammatory cytokines, the VCAM-1 expression levels in the endothelial cells can be significantly elevated (11,12).

VCAM-1 is expressed in vascular endothelial cells, and this expression promotes the adhesion of leukocytes to the endothelial cells. VCAM-1 accelerates the migration of adherent leukocytes along the endothelial surface, and promotes the proliferation of smooth muscle cells; therefore, it is speculated that VCAM-1 may be involved in the pathogenesis of atherosclerosis (13,14). Using animal models, Cybulsky and Gimbrone (15) demonstrated that LDL could promote the expression of VCAM-1 in endothelial cells and that hypercholesterolemia could cause atherosclerosis-related pathophysiological changes in the arteries. In the present study, whole human arterial wall samples were obtained, and the expression of VCAM-1 in the tissue was detected. It was found that the aortic VCAM-1 expression level was upregulated in atherosclerotic patients.

VCAM-1 molecules on the surface of endothelial cells are usually shed into the blood to form soluble proteins (sVCAM-1) (16,17). Due to the difficulty in evaluating VCAM-1 in vascular endothelial tissues, the detection of sVCAM-1 in the blood has typically been used as an indirect indicator for VCAM-1 expression levels (18,19). Hackman *et al* (20) found that the level of sVCAM-1 in the blood was elevated in patients with higher levels of TG. Saidi *et al* (21) additionally showed that, in atherosclerotic patients, an elevated sVCAM-1 level was associated with the activation and damage of endothelial cells; however, these findings would have been of greater significance if they related to the expression of VCAM-1 within the arterial tissues, rather than to the level of the protein in its soluble form. Studies have shown that abnormal lipid metabolism is closely associated with the occurrence and development of atherosclerosis (22–24); therefore, the blood lipid levels and their possible association with VCAM-1 expression were examined in the present study. The results showed that the serum levels of TG, TC, LDL-C, Lp (a) and Apo-B were significantly elevated, whereas the serum HDL-C and Apo-AI levels were significantly decreased, compared with the reference values. Furthermore, the aortic VCAM-1 expression levels were positively correlated with the levels of LDL-C, Lp (a), TC, Apo-B and TG, while a negative correlation was observed between the VCAM-1 and HDL-C levels.

The SYNTAX score is a widely accepted and highly reproducible scoring system that grades the severity and complexity of coronary artery disease (25). Retrospective analyses suggest that the severity of coronary artery disease indicated by the SYNTAX score may be helpful in the selection of revascularization strategies (26–28). The present results showed that there was a significant correlation between the aortic VCAM-1 expression and the SYNTAX score, suggesting that VCAM-1 may participate in the occurrence and development of atherosclerosis. In addition, smoking, diabetes mellitus and hypertension have been recognized as independent risk factors for atherosclerosis (29–31). In the present study it was found that the expression levels of VCAM-1 in the aortic tissues were increased in patients with smoking habits, diabetes mellitus or hypertension, compared with the subjects without these risk factors. These results suggest that cardiovascular risk factors increase the VCAM-1 expression in the arterial tissues, contributing to the occurrence and development of the disease; however, further studies are required to clarify the specific mechanisms.

A number of studies have used animal models to investigate the association between VCAM-1 and the pathogenesis of atherosclerosis (12,32). The establishment of animal models of atherosclerosis is considerably different from the natural pathological process in humans, in terms of disease etiology and pathophysiology. In fact, it is difficult to extrapolate the findings obtained from atherosclerosis animal models to humans. In the present study, the specimens were whole arterial walls, consisting of tunica intima, media and externa. These arterial tissues were used for the investigation of the expression levels of certain inflammatory factors, including nuclear factor-κB, Toll-like receptor 4 and high-mobility group protein B1, in atherosclerotic patients (33–35). Given the fact that aortic tissues were not available from healthy subjects, renal artery tissues from kidney transplant cases were instead used as a control. Despite the fact that the control subjects did not present with any symptoms of atherosclerosis, the possibility of subclinical atherosclerosis could not be ruled out. Another limitation of this study was the small number of atherosclerotic patients. In addition, the SYNTAX scoring system that was used was based on the coronary angiography results, which did not reflect the actual volume of the atherosclerotic plaque. More accurate diagnostic methods, such as intravascular ultrasound and angioscopy, may be more suitable for the evaluation of plaque volume in future studies.

In conclusion, the results of the present study have shown that the expression levels of VCAM-1 in aortic tissues are significantly elevated in atherosclerotic patients and are correlated with blood lipid levels. An upregulated VCAM-1 expression is associated with a higher coronary SYNTAX score, indicating severe coronary artery stenosis. Furthermore, cardiovascular risk factors have also been found to influence the aortic VCAM-1 expression levels.

## Figures and Tables

**Table I.** Blood lipid levels in patients with coronary heart disease.

	Atherosclerosis	Reference values	t-test	P-value
TG (mmol/l)	2.25±1.21	1.13±0.29	5.431	<0.001
TC (mmol/l)	5.13±1.50	4.42±0.79	2.755	0.010
LDL-C (mmol/l)	3.35±1.23	2.59±0.26	3.597	0.002
HDL-C (mmol/l)	1.14±0.33	1.46±0.24	−5.385	<0.001
Lp (a) (mg/dl)	32.31±32.09	14.40±6.64	3.255	0.003
Apo-AI (g/l)	1.08±0.26	1.30±0.15	−4.659	<0.001
Apo-B (g/l)	1.07±0.46	0.85±0.13	2.755	0.024

Results are presented as the mean ± standard deviation. TG, triglycerides; TC, total cholesterol; LDL-C, low-density lipoprotein cholesterol; HDL-C, high-density lipoprotein cholesterol; Lp, lipoprotein; Apo, apolipoprotein.
